# *Desmodesmus pannonicus* Water Extract Inhibits Melanin Synthesis and Promotes Wound Healing

**DOI:** 10.3390/life14121542

**Published:** 2024-11-25

**Authors:** Kazuomi Sato, Yosuke Hiraga, Yuji Yamaguchi, Setsuko Sakaki, Hiroyuki Takenaka

**Affiliations:** 1 Graduate School of Agriculture, Tamagawa University, 6-1-1 Tamagawa-gakuen, Tokyo 194-8610, Japan; 2 MAC Gifu Research Institute, MicroAlgae Corporation, 4-15 Akebono-cho, Gifu 500-8148, Japan; yamaguchi@mac-bio.co.jp (Y.Y.); sakaki@mac-bio.co.jp (S.S.); takenaka@mac-bio.co.jp (H.T.)

**Keywords:** *Desmodesmus*, melanin synthesis, wound healing, tyrosinase

## Abstract

This study investigated the multifaceted benefits of *Desmodesmus pannonicus* water extract across various cell lines, including murine B16F1 melanoma cells, human keratinocyte HaCaT cells, and human follicle dermal papilla cells (HFDPCs), to assess its potential in skin health improvement. Initially, the antioxidant capacity of the extract was evaluated using the ABTS assay, revealing significant radical scavenging activity, indicating strong antioxidative properties. Subsequently, *D. pannonicus* extract showed notable inhibition of α-MSH-enhanced melanin production in B16F1 cells without cell toxicity by suppressing tyrosinase expression. Furthermore, the extract significantly promoted cell migration and enhanced wound healing in HaCaT cells, accompanied by an upregulation of VEGF and MMP mRNA levels, which are crucial for the wound healing process. In addition, we investigated the effect of *D. pannonicus* extract on hair growth-related genes in HFDPCs. Despite a slight reduction in VEGF mRNA levels, an increase in CTGF and HGF1 mRNA levels was observed, alongside a significant down-regulation of TGFβ1, highlighting the extract’s potential to promote hair growth and exhibit antiandrogenic effects. Collectively, these findings demonstrated the therapeutic potential of *D. pannonicus* extract in treating hyperpigmentation, enhancing wound healing, and promoting hair growth, making it a promising candidate for future dermatological applications.

## 1. Introduction

Microalgae are unicellular photosynthetic organisms, gaining attention for their diverse applications and unique characteristics. These organisms are capable of efficient nutrient uptake from water systems [1]. Microalgae are being explored for their potential in energy production, biosynthesis of nutraceuticals, animal feed, and bioremediation [2]. In terms of biofuel sources, microalgae are highly regarded for their rapid growth rate, lipid synthesis capabilities, and carbon dioxide fixation potential [3]. Furthermore, their ability to sequester carbon dioxide from industrial emissions or the atmosphere contributes to the mitigation of greenhouse gas effects. Additionally, Bouyahya et al. reviewed the potential of microalgae as anticancer agents [4]. Microalgae contain flavonoids, phenolic acids, vitamins, and other compounds. These bioactive compounds may contribute to anticancer activity by inducing apoptosis and arresting the cell cycle. Moreover, we reported that the coccolithophore *Pleurochrysis carterae* water extract could inhibit melanin synthesis in B16F1 melanoma cells [5]. In addition, we also found that extract from the cyanobacterium *Nostoc verrucosum* has antioxidative and anti-melanogenic effects [6], which is produced in melanocytes, and serves as a critical protective agent against UV radiation. However, the overproduction of melanin can lead to various hyperpigmentation disorders, such as freckles and melasma. In addition, conditions like solar lentigo and melanoma are associated with both increased melanin production and abnormal proliferation of melanocytes or malignant transformation [7]. Melanogenesis, a process for melanin synthesis, is caused by enzymatic reactions involving tyrosinase, tyrosinase-related protein-1 (TRP-1), and tyrosinase-related protein-2 (TRP-2) [8]. These enzymes play a crucial role in the conversion of the amino acid tyrosine into melanin, the pigment responsible for skin color. The process begins with tyrosinase catalyzing the oxidation of tyrosine to dihydroxyphenylalanine (DOPA) and subsequently to DOPAquinone. DOPAquinone is converted to DOPAchrome and then to 5,6-dihydroxyindole (DHI) or indole 5,6-quinone 2-carboxylic acid (DHICA). TRP-2 catalyzes the oxidation of DHICA. TRP-1 catalyzes the oxidation of DHICA to eumelanin [9].

In cell-based assay screenings, we discovered that *Desmodesmus pannonicus* exhibits an inhibitory effect on melanin synthesis. However, the beneficial effects of *Desmodesmus* on cellular and organismal metabolism remain largely unexplored. Cao et al. reported that polysaccharides extracted from *Desmodesmus armatus* possess antioxidative effects [10]. Furthermore, Ying et al. investigated the effect of a water-soluble extract from *Desmodesmus* sp. YT on human fibroblasts [11]. Their findings indicate that *Desmodesmus* sp. YT mitigates UV-induced oxidative stress by upregulating antioxidant genes, including glutamate-cysteine ligase catalytic subunit (GCLC), NAD(P)H quinone dehydrogenase 1(NQO1), and heme oxygenase-1 (HMOX-1). Consequently, our objective is to evaluate the beneficial effects of *D. pannonicus* water extract for medicinal and cosmetic applications. In this study, we primarily focused on investigating the effect of the extract on melanin synthesis using a cell culture assay system. In addition, we explored its potential effects on wound healing and genes related to hair growth.

## 2. Materials and Methods

### 2.1. Collection and Cultivation of Desmodesmus pannonicus

*D. pannonicus* was collected from Tarui, Gifu Prefecture, Japan. Cells were grown in a C medium containing 15 mg Ca(NO_3_)_2_·4H_2_O, 10 mg KNO_3_, 5 mg β-Na_2_glycerophosphate·5H_2_O, 4 mg MgSO_4_·7H_2_O, 0.01 µg vitamin B12, 0.01 µg biotin, 1 µg thiamine HCl, 0.3 mL PIV metals, and 50 mg tris(hydroxymethil)aminomethane in 99.7 mL distilled water. The PIV metals solution contained 100 mg Na_2_EDTA·2H_2_O, 19.6 mg FeCl_3_·6H_2_O, 3.6 mg MnCl_2_·4H_2_O, 2.2 mg ZnCl_2_, 0.4 mg CoCl_2_·6H_2_O, and 0.25 mg Na_2_MoO·4H_2_O in 1 L distilled water. The cells were grown in a cylindrical acrylic tank (200 L, Φ5 cm × 10 cm) at a temperature of 23 ± 2 °C and a continuous illumination of 150 μmol photon m^−2^ s^−1^ for 10 days. The cells were harvested by centrifugation and immediately dehydrated by freeze-drying. The dried algal powder was extracted by stirring in hot water at 85–90 °C for one hour. Following this, the mixture was centrifuged at 10,000 rpm for 15 min. Then, the supernatant was filtered through a GA100 glass filter (ADVANTEC, Tokyo, Japan). The filtrate obtained was concentrated and then was filtered through a 0.22 µm filter (Merck Millipore, Burlington, MA, USA) for sterilization.

The sterilized hot water extract of *D. pannonicus* was stored at −20 °C until it was used in this study.

### 2.2. Cell Culture

B16F1 melanoma (provided by the Riken BioResource Center, Ibaraki, Japan) and HaCaT cells (Cosmo Bio Co., Ltd., Tokyo, Japan) were cultured in Dulbecco’s modified Eagle’s medium (DMEM; Sigma-Aldrich, St. Louis, MO, USA) supplemented with 10% fetal bovine serum (FBS), 50 U/mL penicillin, and 100 µg/mL streptomycin. Cultures were maintained at 37 °C in a humidified atmosphere containing 5% CO_2_. HFDPCs (PromoCell, Heidelberg, Germany) were cultured in Follicle Dermal Papilla Cell Growth Medium (PromoCell).

### 2.3. Assay of 2,2′-Azino-bis (3-ethylbenzothiazoline-6-sulfonic acid (ABTS) Radical Scavenging Activity

The ABTS radical scavenging activity was determined using the method described by Re et al., with some modifications [12]. For the assay, 180 µL of the ABTS radical solution, which was prepared according to standard procedures, was mixed with 20 µL of *D. pannonicus* extracts at various concentrations. The mixture was then incubated at room temperature in the dark for 30 min. After the incubation period, the absorbance of each sample was measured at a wavelength of 732 nm using a spectrophotometer. The ABTS radical scavenging activity was quantified based on the reduction in absorbance compared to the control. The percentage of ABTS radical scavenging activity was calculated using the following formula:

ABTS radical scavenging activity (%) = (1 − Asample/Acontrol) × 100.

### 2.4. Cell Viability and Proliferation

To investigate the effects of *D. pannonicus* extract on cell viability, we performed the 3-(4.5-dimethylthiazol-yl)-2,5-diphenyltetrazolium bromide (MTT) assay. B16F1, HaCaT, and HFDPCs were plated in 24-well plates at a density of 1 × 10^4^ cells/well. After 24 h of incubation, these cells were treated with *D. pannonicus* extract for an additional 24 h. After treatment, 20 µL of 5 mg/mL MTT (Sigma) was added and incubated for 3 h and 30 min at 37 °C. Finally, the precipitate formazan was dissolved with DMSO and the absorbance was measured at 590 nm using a microplate reader.

To assess cell proliferation, B16F1 melanoma cells were seeded in a 60 mm dish at a density of 1 × 10^5^ cells per dish. Following a similar 24 h of incubation, the cells were treated with D. pannonicus extract for 3 days in the presence of α-MSH (10 nM). After treatment, the cells were washed with phosphate-buffered saline (PBS) and detached using trypsin/EDTA solution (Life Technologies, Carlsbad, CA, USA). The number of viable cells, indicative of cell proliferation, was then quantified using trypan blue.

### 2.5. Melanin Content

The melanin content was measured as previously described [13]. For this assay, B16F1 melanoma cells were treated with *D. pannonicus* extract for 3 days in the presence of α-MSH. Following the treatment, the cells were detached using trypsinization, and their numbers were determined using a cytometer. After counting, the cells were washed and subsequently lysed in boiling 2 M NaOH. The melanin content of the lysate was then assessed using spectrophotometry. The absorbance was measured at a wavelength of 405 nm.

### 2.6. Activities of Mushroom Tyrosinase

The direct effects of *D. pannonicus* on monophenolase and diphenolase activities were assessed using a cell-free assay system with mushroom tyrosinase. For the monophenolase assay, L-tyrosine (Fujifilm Wako, Osaka, Japan) served as the substrate. First, 80 µL of 0.1 M phosphate buffer (pH 6.8), 80 µL of 1.25 mM L-tyrosine, and 20 µL of distilled water or *D. pannonicus* water extract were mixed. Subsequently, 20 µL of 500 units/mL mushroom tyrosinase (Sigma) in aqueous solution was added, and the reaction was conducted at 25 °C. Enzyme activity was monitored at 475 nm every 30 s for 6 min using a microplate reader (SH9000 Lab, Hitachi High-Tech Science Co., Tokyo, Japan). The percentage of monophenolase activity was calculated as follows:

100 − [(A − B)/A × 100],

where ‘A’ represents the change in the absorbance of the control sample between incubation times of 3.0 and 6.0 min, and ‘B’ indicates the change in the absorbance of the test sample over the same period.

The diphenolase activity assay was performed using L-3,4-dihydroxyphenylalanine (L-DOPA, Sigma) as the substrate. Initially, 50 µL of 0.5 mg/mL L-DOPA solution, 20 µL of *D. pannonicus* water extract, and 110 µL of 0.1 M phosphate buffer (pH 6.8) were mixed. Following the pre-incubation, 20 µL of 1000 units/mL mushroom tyrosinase in aqueous solution was added, and the reaction’s progress was monitored at 475 nm every 15 s for 1 min. The percentage of tyrosinase activity was calculated using the following formula:

100 − [(A − B)/A × 100],

where ‘A’ represents the difference in the absorbance of the control sample between an incubation time of 0.5 and 1.0 min, and ‘B’ represents the difference in the absorbance of the test sample over the same incubation period.

### 2.7. Intracellular Tyrosinase Activity Analysis (DOPA Staining)

The assessment of tyrosinase activity using DOPA staining was conducted following a modified version of the previously reported protocol [13]. B16F1 melanoma cells were stimulated by α-MSH (10 nM) and subsequently treated with or without *D. pannonicus* extract for 3 days. For the detection of active isoforms of tyrosinase, cell lysates were prepared in a buffer composed of 0.1 M sodium phosphate (pH 6.8), 1% Triton X-100, 1 mM phenylmethylsulfonyl fluoride, along with 10 µg/mL each of aprotinin and leupeptin, while deliberately omitting mercaptoethanol and heat treatment. These lysates were then subjected to separation via 7.5% SDS-PAGE. Subsequently, gels containing tyrosinase bands were placed in a flat-bottomed container with 200 mL of sodium phosphate buffer and equilibrated at room temperature with gentle shaking. After 30 min, the buffer was replaced with a fresh buffer. Following a second rinse, the gels were immersed in 200 mL of a staining solution containing sodium phosphate buffer supplemented with 0.5 mg/mL L-DOPA. This was then incubated in a dark environment at 37 °C for 2 h. The presence of tyrosinase activity was indicated by the emergence of dark melanin bands in the gels.

### 2.8. Western Blotting

To investigate the effect of *D. pannonicus* extract on the expression of melanogenic proteins, we conducted a Western blotting analysis. B16F1 melanoma cells were treated with *D. pannonicus* extract, both in the presence and absence of α-MSH. Following treatment, the cells were lysed using RIPA lysis buffer. The whole-cell lysates were then subjected to SDS-PAGE. After electrophoresis, the resolved proteins were transferred onto PVDF membranes (Merck Millipore). After blocking with 5% skimmed milk in PBS containing 0.1% Tween 20, the membranes were probed overnight with tyrosinase, TRP-1, TRP-2, Mitf, and MC1R antibodies (all obtained from Santa Cruz Biotechnology, Dallas, TX, USA) at 4 °C and further incubated with horseradish peroxidase-conjugated secondary antibodies. The bound antibodies were detected via chemiluminescence using an ImmunoStar zeta or ImmunoStar LD (Fujifilm Wako, Osaka, Japan) following the manufacturer’s instructions.

### 2.9. Quantitative RT-PCR

To investigate the effects of *D. pannonicus* on gene transcription, we treated B16F1, HaCaT, and HFDPCs with *D. pannonicus* extract. Following the treatment, total RNA was extracted from the cells using ISOGEN II (Nippon Gene, Tokyo, Japan). The extracted RNA was converted to cDNA using AMV Reverse Transcriptase (TaKaRa, Shiga, Japan). The sequence of specific primers used is shown in Table 1. PCR amplification was performed as described in our previous report [6] using a 7500 Fast Real-Time PCR system (Thermo Fisher Scientific, Waltham, MA, USA). The relative expression levels of the target genes were normalized to that of GAPDH and calculated using the 2^−ΔΔCt^ method. All experiments were performed in triplicate.

### 2.10. Scratch Wound-Healing Assay

To evaluate the wound-healing potential of *D. pannonicus*, we conducted a scratch wound-healing assay as previously described [14]. HaCaT cells (1 × 10^5^ cells per well) were seeded into 24-well plates and cultured until they formed a monolayer of 90–100% confluence. A mechanical scratch was then created on the cell monolayer using a 200 µL pipette tip. To remove cellular debris, the cells were washed three times with PBS. Subsequently, the cells were treated with increasing doses of *D. pannonicus* extract and incubated for an additional 30 h. Wound closure was monitored using the EVOS FL imaging system (Thermo Fisher Scientific), with wounds photographed at the same location at 0, 24, and 30 h postscratch. The reduction in the wound area over time, compared to the initial wound size, was quantified using ImageJ software (version 1.54g).

### 2.11. Statistical Analysis

Statistical significance was analyzed with Dunnett’s test using the GraphPad Prism 10 software (GraphPad Software, Inc., La Jolla, CA, USA). All numerical data are presented as the mean ± SEM of experiments performed at least in triplicate. Results with *p* < 0.05 and *p* < 0.01 were considered statistically significant.

## 3. Results

### 3.1. Antioxidant Activity

**Figure 1:**
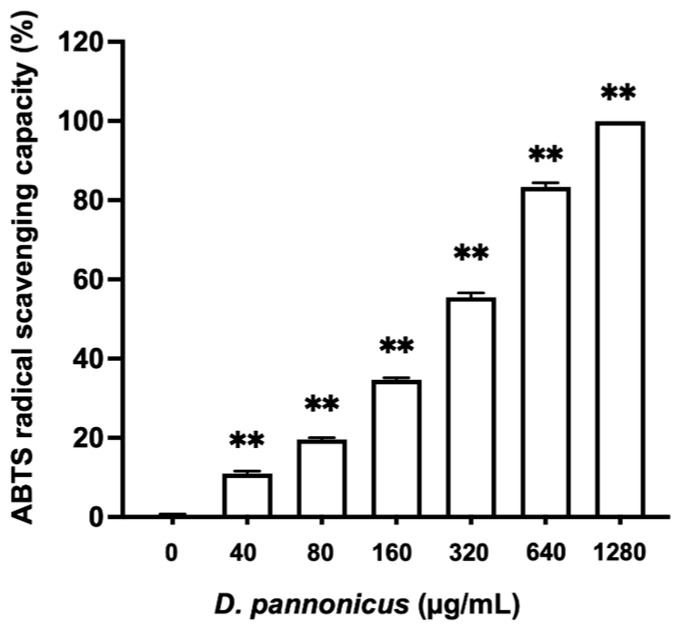
Antioxidant activity of *D. pannonicus* extract. The antioxidant activity of *D. pannonicus* extract was measured using an ABTS radical scavenging assay. Results are represented as ABTS radical scavenging activity (%). The data are shown as mean ± SEM of at least three separate experiments. ** *p* < 0.01 compared with the control group.

The ABTS assay was performed to assess the radical scavenging activities of *D. pannonicus*. The results demonstrated a clear dose-dependent increase in the radical scavenging activity of *D. pannonicus* extracts (Figure 1). The extracts were tested across a range of concentrations, from 40 to 1280 µg/mL. At lower concentrations, the extracts exhibited moderate radical scavenging activity. At the highest concentration tested, 1280 µg/mL, the extract exhibited almost complete radical scavenging activity.

### 3.2. Effect of D. pannonicus on Cell Viability and Proliferation

**Figure 2:**
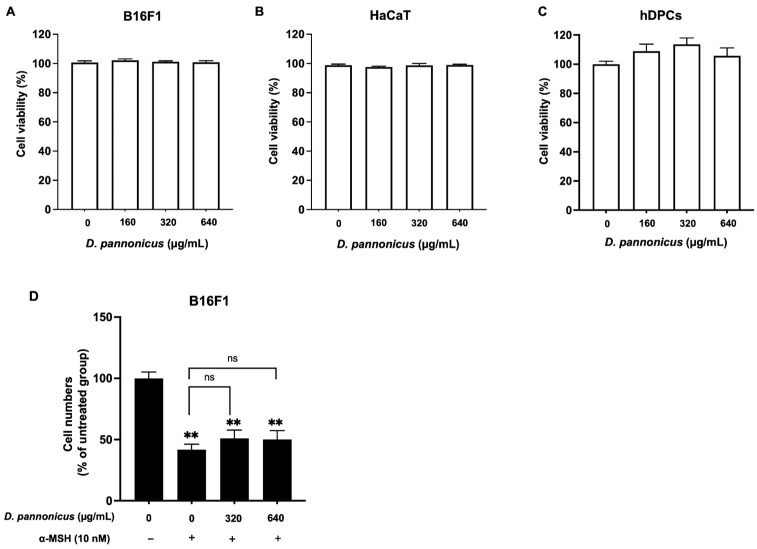
Assessing the effect of *D. pannonicus* on cell viability and proliferation. (**A**) B16F1 melanoma cells, (**B**) HaCaT cells, and (**C**) human follicle dermal papilla cells (HFDPCs) were treated with *D. pannonicus* extract for 24 h. After treatment, cell viability was assessed using the MTT assay. (**D**) B16F1 melanoma cells were treated with *D. pannonicus* extract in the presence of α-MSH for 3 days. After treatment, the trypan blue exclusion test was performed, and the cell proliferation rate was assessed. The data are shown as mean ± SEM of at least three independent experiments. Statistical significance is indicated as ** *p* < 0.01.

In the cell-based assay, we first evaluated the effect of *D. pannonicus* extract on cell viability using B16F1, HaCaT, and HFDPCs. As shown in Figure 2A–C, treatment with *D. pannonicus* extract did not adversely affect the viability of any of the cell lines tested. Furthermore, we assessed the effect of *D. pannonicus* extract on cell proliferation in the presence of α-MSH. Figure 2D shows that cells treated with α-MSH alone exhibited reduced proliferation compared with the untreated control. However, the addition of *D. pannonicus* extract to α-MSH-treated cells did not further decrease cell proliferation. These findings suggest that *D. pannonicus* extract does not inhibit cell proliferation, even in the context of α-MSH-induced suppression of cell growth.

### 3.3. D. pannonicus Extract Inhibits Melanin Synthesis

**Figure 3:**
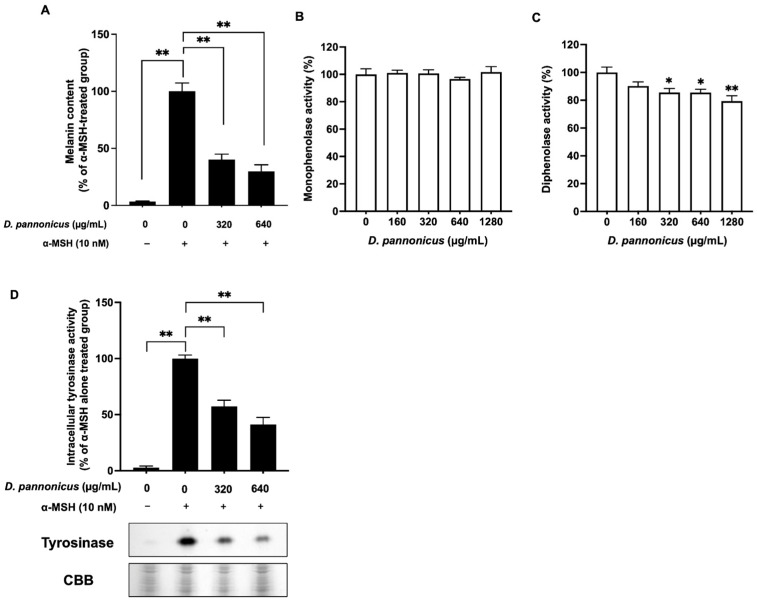
Effect of *D. pannonicus* extract on melanin synthesis in B16F1 melanoma cells. (**A**) The cells were treated with or without *D. pannonicus* and stimulated with α-MSH for 3 days, and the melanin content was measured. (**B**) Monophenolase and (**C**) diphenolase activities were assessed using mushroom tyrosinase in the presence of various concentrations of *D. pannonicus* extract. (**D**) The effect of *D. pannonicus* extract on the intracellular active form of tyrosinase was evaluated. B16F1 melanoma cells were treated with *D. pannonicus* extract for 3 days in the presence of 10 nM α-MSH. After treatment, the cell lysates were subjected to SDS-PAGE followed by DOPA staining to visualize the tyrosinase activity. The intensity of the tyrosinase bands was quantified using ImageJ software. Statistical significance is indicated as * *p* < 0.05 and ** *p* < 0.01.

To investigate the effect of *D. pannonicus* extract on melanogenesis, B16F1 melanoma cells stimulated with α-MSH were treated with the extract for 3 days. α-MSH is known to elevate intracellular cAMP levels, thereby stimulating the melanin synthesis pathway [15]. As shown in Figure 3, cells treated solely with α-MSH exhibited increased melanin content, confirming α-MSH’s stimulatory effect on melanogenesis. Conversely, after treatment with *D. pannonicus* extract in the presence of α-MSH, a notable suppression in melanin synthesis was observed. This result indicated that *D. pannonicus* extract effectively inhibited the melanin synthesis pathway activated by α-MSH in B16F1 melanoma cells.

Given the hypothesis that *D. pannonicus* might inhibit melanin synthesis through the suppression of tyrosinase enzyme activities, we evaluated both monophenolase and diphenolase activities using mushroom tyrosinase. As shown in Figure 3B, the monophenolase activity remained unaffected by *D. pannonicus* treatment. Conversely, a slight inhibition of the diphenolase activity was observed upon treatment (Figure 3C). However, the inhibition of mushroom tyrosinase by *D. pannonicus* extract was not deemed to significantly contribute to the observed suppression of melanin synthesis. Therefore, a DOPA staining assay was conducted to assess the intracellular active form of tyrosinase in *D. pannonicus*-treated B16F1 melanoma cells. As shown in Figure 3D, the *D. pannonicus* extract markedly inhibited α-MSH-enhanced tyrosinase activity, indicating that *D. pannonicus* may suppress melanin synthesis through the inhibition of tyrosinase protein expression and/or gene transcription.

### 3.4. The Effects of D. pannonicus Extract on Melanogenic Protein Expression

**Figure 4:**
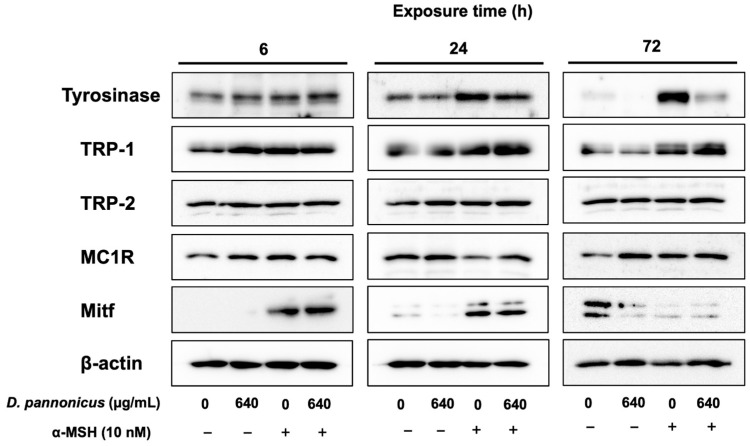
Melanogenic protein expression in B16F1 melanoma cells treated with *D. pannonicus* extract. B16F1 melanoma cells were treated with *D. pannonicus* extract for 6, 24, and 72 h in the presence of 10 nM α-MSH. After treatment, total cell lysates were prepared, and Western blot analysis was performed to assess melanogenic protein expression.

We explored the effects of *D. pannonicus* extract on the expression of melanogenic proteins in B16F1 melanoma cells under various treatment durations: 6, 24, and 72. The cells were treated in four different conditions: untreated control, extract only, α-MSH only, and a co-treatment with α-MSH and extract. These experimental conditions enabled us to assess the effect of the extract both in the presence and absence of α-MSH stimulation. As shown in Figure 4, for 72 h of treatment of *D. pannonicus* extract significantly inhibited the α-MSH-enhanced tyrosinase protein level. While a faint tyrosinase band was visible in cells without α-MSH treatment, the tyrosinase expression was further suppressed in cells that were treated with the extract. Similar results were observed in the cells that were treated for 24 h. However, in the 6 h treatment group, tyrosinase expression did not show a significant change. Regarding TRP-1 expression, although α-MSH stimulated enhanced protein levels, *D. pannonicus* extract did not inhibit this protein level. For TRP-2 and MC1R proteins, our results indicated that *D. pannonicus* extract had no significant effect on their expression across all treatment durations. Mitf protein levels were enhanced after α-MSH stimulation for 6 and 24 h. However, *D. pannonicus* did not inhibit α-MSH-enhanced Mitf protein levels. These results imply that *D. pannonicus* inhibits melanin synthesis via the suppression of tyrosinase protein expression.

### 3.5. D. pannonicus Suppressed the Tyrosinase mRNA Levels

**Figure 5:**
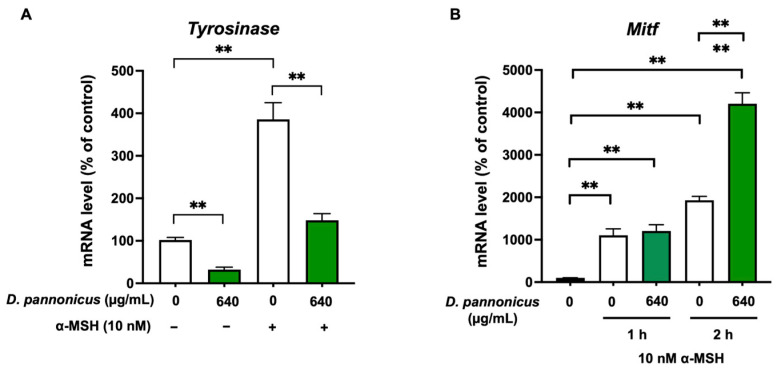
Effect of *D. pannonicus* extract on melanogenic gene transcription in B16F1 melanoma cells. (**A**) B16F1 melanoma cells were treated with *D. pannonicus* extract and subsequently stimulated with 10 nM α-MSH for 24 h. (**B**) Cells were pretreated with *D. pannonicus* extract for 1 h, before the addition of α-MSH (10 nM), followed by incubation for either 1 or 2 h in the presence of α-MSH. Total RNA was extracted, and cDNA was synthesized. Quantitative PCR was performed using specific primers for the *tyrosinase* and *Mitf* genes. The data are shown as mean ± SEM of at least three independent experiments. Statistical significance is indicated as ** *p* < 0.01.

We performed qRT-PCR analysis to investigate the influence of *D. pannonicus* on the gene expression of *tyrosinase* and *Mitf* in B16F1 cells. As shown in Figure 5A, the cells were treated with various combinations of *D. pannonicus* and α-MSH for a duration of 24 h. Our results revealed that *D. pannonicus* extract led to a significant suppression of *tyrosinase* mRNA levels compared with the untreated control. This effect was also observed in cells in the presence of α-MSH. Further analysis focused on the expression of *Mitf* mRNA under short-term α-MSH stimulation, considering the sensitivity of *Mitf* to external stimuli. As shown in Figure 5B, α-MSH stimulation for both 1 and 2 h resulted in a marked increase in *Mitf* mRNA levels. Interestingly, when cells were pretreated with *D. pannonicus* extract, there was a significant reduction in mRNA levels following 1 h of α-MSH stimulation. In contrast, a 2 h α-MSH stimulation in the presence of *D. pannonicus* extract appeared to enhance *Mitf* mRNA levels.

### 3.6. Wound Healing Effect of D. pannonicus Extract

**Figure 6:**
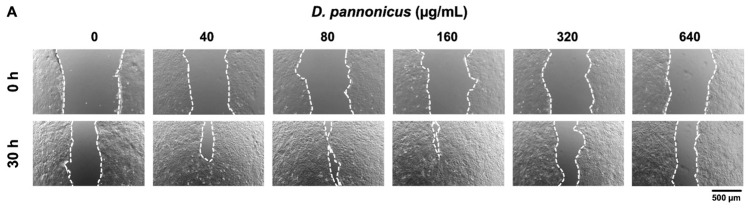

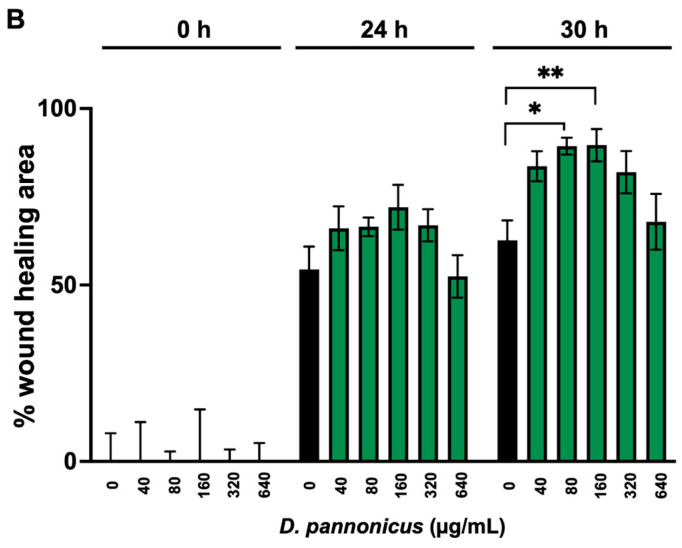
The wound healing effect of *D. pannonicus* on HaCaT cells. (**A**) Representative images of HaCaT cells treated with *D. pannonicus* extract following a scratch wound. The cells were photographed at 0, 24, and 30 h postscratch to monitor wound closure over time. (**B**) Percentages of wound closure at various time points, calculated as the relative reduction in the wound area compared to the initial wound area at 0 h, which is set as 0% (with complete closure represented as 100%). The data are shown as mean ± SEM of at least three independent experiments. Statistical significance is indicated as * *p* < 0.05 and ** *p* < 0.01.

We investigated the potential wound-healing activity of *D. pannonicus* extract on HaCaT cells using a scratch wound-healing assay. This assay quantitatively measures the progressive closure of wounds, assessing the extent of healing by calculating the percentage increase in the closed area as cells migrate and proliferate over time. As shown in Figure 6A, treatment with *D. pannonicus* extract markedly accelerated wound closure compared with the control. Furthermore, Figure 6B demonstrated that *D. pannonicus* extract significantly enhanced wound healing in HaCaT cells at concentrations of 80 and 160 µg/mL. However, at the higher concentration of 640 µg/mL, the wound area remained as open as in the control group, indicating no significant wound-healing effect at this concentration.

### 3.7. Effect of D. pannonicus Extract on Gene Expression in HaCaT Cells

**Figure 7:**
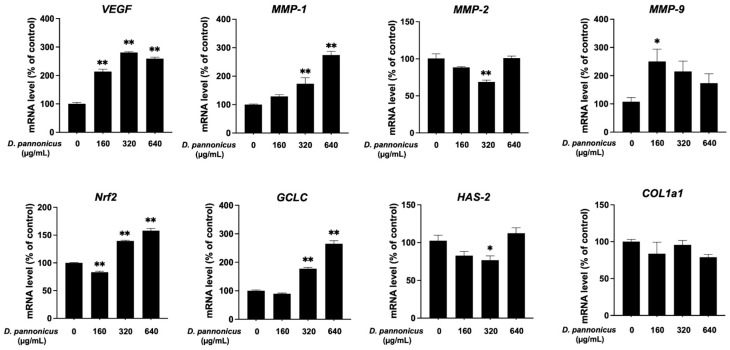
Effect of *D. pannonicus* extract on gene expression in HaCaT cells. HaCaT cells were treated with various concentrations of *D. pannonicus* extract for 48 h. After treatment, the total RNA was extracted, and cDNA was synthesized. Quantitative PCR was then performed using specific primers for *VEGF*, *MMP-1*, *MMP-2*, *MMP-9*, *Nrf2*, *GCLC*, *HAS-2*, and *COL1a1*. The data are shown as mean ± SEM of at least three independent experiments. Statistical significance is indicated as * *p* < 0.05 and ** *p* < 0.01.

To explore the mechanism by which *D. pannonicus* extract accelerates the wound-healing process, we assessed the mRNA transcription levels of genes related to wound healing. After treating HaCaT cells with *D. pannonicus* extract for 48 h, total RNA was extracted, and mRNA expression levels were evaluated using quantitative RT-PCR analysis. The vascular endothelial cell growth factor (*VEGF*), a critical angiogenic factor in wound closure, showed a significant increase in RNA levels following treatment with *D. pannonicus* (Figure 7). In addition, we examined the expression of matrix metalloproteinases (MMPs), which facilitate the migration of fibroblasts and keratinocytes essential for wound healing. The mRNA levels of *MMP-1* and *MMP-9* were elevated after treatment, whereas *MMP-2* levels decreased following treatment with *D. pannonicus* at a concentration of 320 µg/mL for 48 h. Increases in the expression of antioxidant-related genes, such as *Nrf-2* and *GCLC*, were also observed. Conversely, the expression of hyaluronan synthase 2 (*HAS-2*) was slightly reduced after treatment.

### 3.8. Effect of D. pannonicus Extract on Hair Growth-Related Gene Expression in HFDPCs

**Figure 8:**
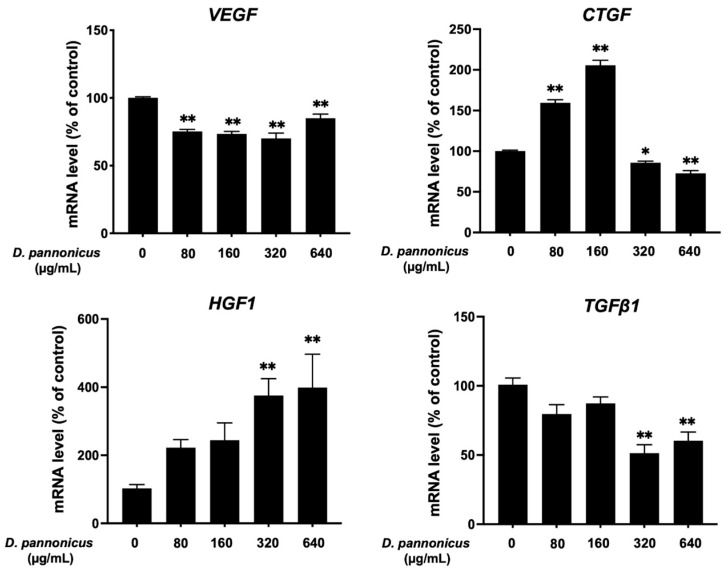
Effect of *D. pannonicus* extract on hair growth-related genes in HFDPCs. HFDPCs were treated with various concentrations of *D. pannonicus* extract for 48 h. After treatment, the total RNA was extracted, and cDNA was synthesized. Quantitative PCR was then performed using specific primers for *VEGF*, *CTGF*, *HGF1*, and *TGFβ1*. The data are shown as mean ± SEM of at least three independent experiments. Statistical significance is indicated as * *p* < 0.05 and ** *p* < 0.01.

HFDPCs were treated with *D. pannonicus* extract for 48 h, followed by quantitative real-time PCR (qRT-PCR) analysis to assess the expression levels of four genes known to be involved in the hair cycle or growth: *VEGF*, *CTGF*, *HGF1*, and *TGFβ1* (Figure 8). *VEGF* showed a slight decrease in expression after treatment with the extract. In contrast, a significant upregulation was observed in the expression levels of the *CTGF* and *HGF1* genes, indicating a positive response to *D. pannonicus* treatment in promoting factors associated with hair growth. However, at higher concentrations (320 and 640 µg/mL), the CTGF mRNA level was slightly decreased. Conversely, the *TGFβ1* gene exhibited a notable decrease in expression, suggesting that the *D. pannonicus* extract might contribute to the prevention of hair loss.

## 4. Discussion

In this study, we investigated the effects of *D. pannonicus* water extract on melanin synthesis, wound healing, and hair cycle-related genes across several cell lines, including B16F1 melanoma cells, HaCaT cells, and human follicle dermal papilla cells (HFDPCs), to assess the beneficial properties of *D. pannonicus*. Initially, we conducted an ABTS assay, which revealed that the extract possesses significant radical scavenging capacity. This finding is consistent with a previous study by Cao et al., who reported that polysaccharides from *Desmodesmus armatus* exhibited strong radical scavenging activity [10]. Therefore, our results provide evidence that *Desmodesmus pannonicus* and potentially other *Desmodesmus* species may have antioxidative activities.

Furthermore, we performed the MTT assay to evaluate cell viability following treatment with *D. pannonicus*. Our findings indicate that *D. pannonicus* did not induce cell death in any of the cell lines tested.

**Figure 9:**
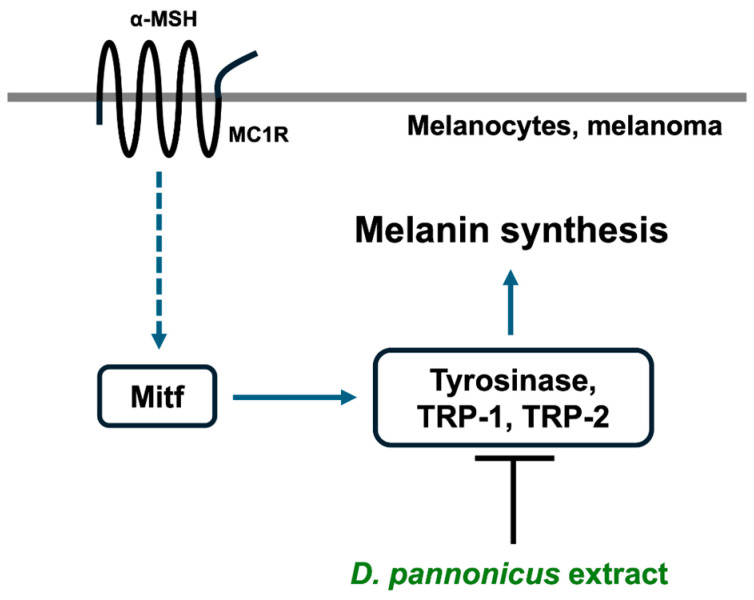
Schematic representation of the melanin synthesis pathway.

Skin color is determined by melanin synthesis in the basal layer of the skin. Abnormal melanogenesis can lead to various esthetic issues and hyperpigmentation disorders [16]. We assessed the potential of *D. pannonicus* extract as an antimelanogenic agent using B16F1 melanoma cells. Our findings revealed that the extract inhibited α-MSH-enhanced melanin content, yet it did not strongly inhibit the monophenolase and diphenolase activities of mushroom tyrosinase. This suggests that *D. pannonicus* may suppress melanin synthesis through the down-regulation of tyrosinase protein expression or other melanogenic protein expressions. Consequently, we performed tyrosinase zymography, Western blotting, and quantitative RT-PCR analysis on *D. pannonicus*-treated B16F1 melanoma cells. Notably, tyrosinase protein levels were significantly reduced following treatment, whereas the levels of other melanogenic proteins such as TRP-1, TRP-2, Mitf, and MC1R remained unchanged. This indicates that the mechanism of melanin synthesis inhibition by *D. pannonicus* extract may primarily involve the down-regulation of tyrosinase protein expression (Figure 9).

Wound healing is a multifaceted process involving the repair of damaged tissue [17]. To investigate the effects of *D. pannonicus* extract on this process, we conducted a scratch wound-healing assay using HaCaT cells. Our findings demonstrate that the extract significantly promoted cell migration and subsequently enhanced wound healing. Furthermore, quantitative RT-PCR analysis was performed to examine the expression of wound healing-related genes following treatment with *D. pannonicus*. VEGF plays a pivotal role in inducing angiogenesis and facilitating wound repair [18]. Additionally, matrix metalloproteinases (MMPs), known to be involved in the wound healing process, were evaluated. Our data revealed the upregulation of *MMP-1* and *MMP-2*, while *MMP-2* expression was slightly inhibited. This observation is consistent with the findings of Nguyen et al., who reported that the role of MMP-2 in wound healing is negligible [19].

Moreover, we explored the effect of *D. pannonicus* extract on antioxidant-related genes, focusing on erythroid-derived-2-like 2 (*Nrf2*) and the glutamate-cysteine ligase catalytic subunit (*GCLC*). *Nrf2*, a key transcription factor, activates a series of genes that protect cells against oxidative stress [20]. *GCLC*, a downstream target of *Nrf2*, plays a crucial role in augmenting glutathione levels, which is essential for the cellular detoxification process [21,22]. The treatment significantly upregulated these genes in HaCaT cells, underscoring *D. pannonicus*’s potential to enhance the antioxidant defense mechanism. This finding is consistent with research by Ying et al. [11], who reported the antioxidative gene enhancement by *Desmodesmus*, including *GCLC*. Our results corroborate the existing data, highlighting the capacity of *Desmodesmus* to boost antioxidative gene expression, thereby contributing to its wound-healing efficacy.

Finally, we evaluated the effect of *D. pannonicus* extract on hair growth-related genes in HFDPCs. It is known that VEGF plays a protective role in human follicle cells with its expression notably diminished in the bald scalps of patients suffering from hair follicle diseases [23,24]. Contrary to expectations, *D. pannonicus* extract slightly reduced *VEGF* mRNA levels in HFDPCs under our experimental conditions. Recent studies, such as those conducted by Albalawi et al., have suggested that connective tissue growth factor (CTGF) may prevent hair loss [25]. In our experimental conditions, *D. pannonicus* treatment significantly increased *CTGF* mRNA levels at lower concentrations, while down-regulation was observed at higher concentrations. In addition, it has been reported that hepatocyte growth factor (HGF) contributes to hair follicle morphogenesis and the extension of the anagen phase [26]. Consistent with this, we observed an increase in *HGF* mRNA levels following treatment with *D. pannonicus*. TGFβ1, recognized as a biomarker for hair loss and closely associated with androgenetic alopecia, was significantly down-regulated by our treatment [27,28]. Collectively, these findings suggest that *D. pannonicus* extract may promote the extension of the anagen phase, thereby enhancing hair growth and exhibiting antiandrogenic effects.

In conclusion, our comprehensive study demonstrated the multiple benefits of *D. pannonicus* water extract across several cellular models. We confirmed that the extract inhibited melanin synthesis without affecting cell viability through the down-regulation of tyrosinase expression. Furthermore, the extract’s promotion of wound healing through the upregulation of *VEGF* and *MMP* mRNA levels highlights its potential use in dermatological treatments for skin repair and regeneration. Our findings on hair growth-related genes revealed that the extract has the potential to promote hair growth by modulating key biomarkers. Collectively, these results highlight *D. pannonicus*’s promising applications in cosmetics and therapeutic fields. Future studies should focus on isolating and characterizing the active compounds within the extract that contribute to these effects. Further, in vivo studies and clinical trials will also be essential to validate the efficacy and safety of *D. pannonicus* extract in human applications. These efforts could pave the way for the development of algae-derived products in the cosmetics and medical industries.

## Figures and Tables

**Table 1:** List of primers used for the real-time polymerase chain reaction analysis.

Gene	Forward Primer	Reverse Primer
*Tyrosinase* (mouse)	TTGCCACTTCATGTCATCATAGAATATT	TTTATCAAAGGTGTGACTGCTATACAAAT
*Mitf* (mouse)	CGCCTGATCTGGTGAATCG	CCTGGCTGCAGTTCTCAAGAA
*GAPDH* (mouse)	CGTCCCGTAGACAAAATGGT	TTGATGGCAACAATCTCCAC
*VEGF* (human)	CTTCTGAGTTGCCCAGGAGA	GGATGGAGGAAGGTCAACCA
*MMP-1* (human)	GGGAGATCATCGGGACAACTC	TGAGCATCCCCTCCAATACC
*MMP-2* (human)	TAGCAGCGGAACAAGGAG	AAACGGGAACCAGGACAC
*Nrf2* (human)	AACCAGTGGATCTGCCAACTACTC	CTGCGCCAAAAGCTGCAT
*GCLC* (human)	GATGCTGTCTTGCAGGGAATG	AGCGAGCTCCGTGCTGTT
*HAS-2* (human)	TGGATGACCTACGAAGCGATTA	GCTGGATTACTGTGGCAATGAG
*COL1a1* (human)	AGGACAAGAGGCATGTCTGGTT	TTGCAGTGGTAGGTGATGTTCTG
*CTGF* (human)	GTTTGGCCCAGACCCAACTA	GGCTCTGCTTCTCTAGCCTG
*HGF1* (human)	AGAAATGCAGCCAGCATCAT	CACATGGTCCTGATCCAATC
*TGFβ1* (human)	GCCCTGGACACCAACTATTG	GTCCAGGCTCCAAATGTAGG
*GAPDH* (human)	CTTTGGTATCGTGGAAGGACTC	GTAGAGGCAGGGATGATGTTCT

## Data Availability

Data are available upon request.
